# Predictors of recurrence in breast cancer patients with pathological partial response

**DOI:** 10.1590/1806-9282.20231215

**Published:** 2024-04-22

**Authors:** Fadime Didem Can Trabulus, Mehmet Ali Nazli, Esra Arslan, Ozlem Mermut, Fatih Dal, Bulent Akce, Riza Umar Gursu, Esra Canan Kelten Talu, Jacqueline Nur Adira Couteau

**Affiliations:** 1 Bahçeşehir University, School of Medicine, Goztepe Medical Park Hospital, Department of General Surgery – İstanbul, Turkey.; 2 University of Health Sciences, Istanbul Training and Education Hospital, Department of Radiology – İstanbul, Turkey.; 3 University of Health Sciences, Istanbul Training and Education Hospital, Department of Nuclear Medicine – İstanbul, Turkey.; 4 University of Health Sciences, Istanbul Training and Education Hospital, Department of Radiation Oncology – İstanbul, Turkey.; 5 University of Health Sciences, Istanbul Training and Education Hospital, Department of General Surgery – İstanbul, Turkey.; 6 University of Health Sciences, Istanbul Training and Education Hospital, Department of Medical Oncology – İstanbul, Turkey.; 7 University of Health Sciences, İzmir Faculty of Medicine, Department of Pathology – İzmir, Turkey.; 8 Bahçeşehir University, School of Medicine – İstanbul, Turkey.

**Keywords:** Breast cancer, Pathologic complete response, Neoadjuvant chemotherapy, Disease free survival, Locoregional neoplasm recurrences

## Abstract

**OBJECTIVE:**

Patients with residual disease after neoadjuvant chemotherapy have a relative risk of developing recurrence. This study investigates the risk factors for recurrence in locally advanced breast cancer patients with residual disease and evaluates survival analysis.

**METHODS:**

This is a retrospective, single-center study. Breast cancer patients who failed to achieve a pathological complete response after neoadjuvant chemotherapy were included. Demographic, clinicopathological, and treatment characteristics were evaluated to identify predictive factors of recurrence and survival analysis.

**RESULTS:**

We included 205 patients in this study. After a median of 31 months of follow-up, 10 patients died, and 20 developed distant metastasis. Disease-free survival and disease-specific survival were 73.8% and 83.1%, respectively. Lymphovascular invasion and non-luminal subtype were independent predictors of locoregional recurrence. In situ carcinoma, lymphovascular invasion, ypTIII stage, and non-luminal molecular subtypes were independent predictors of disease-free survival. The only independent factor affecting disease-specific survival was cNII–III. The number of involved lymph nodes was an independent predictor of disease-free survival in patients without complete axillary response.

**CONCLUSION:**

Factors affecting disease-specific survival and disease-free survival were cNII–III and the number of involved lymph nodes, respectively. Patients with non-luminal, large residual tumors with in situ carcinoma, lymphovascular invasion, clinically positive axilla, and residual nodal involvement have a high relative risk for recurrence and may benefit from additional treatments.

## INTRODUCTION

Neoadjuvant chemotherapy (NAC) is the standard approach for managing locally advanced breast cancer (LABC) (stages IIB and III)^1,2^. NAC downstages tumors by allowing breast-conserving surgery (BCS) instead of mastectomy and avoids axillary lymph node dissection (ALND)-associated lymphedema by allowing sentinel lymph node biopsy (SLNB)^1-3^. NAC is recommended for triple-negative breast cancer (TNBC) and human epidermal growth factor receptor 2 (HER2)-positive cancer because these subtypes have higher rates of pathological complete response (pCR)^1-5^.

In LABC patients, pCR following NAC can serve as a surrogate marker of treatment efficacy^1,3^. However, not all patients achieve pCR and may develop locoregional recurrence (LRR)^6^. Patients with a partial response may avoid recurrence by the addition of regimens that will sensitize their tumors to ongoing chemotherapy^7^. Therefore, identifying patients at high risk of recurrence is critical for early intervention. This study aimed to identify factors that predict recurrence in LABC patients who failed to achieve pCR after NAC and to conduct a survival analysis.

## METHODS

### Study design and patient selection

This retrospective study was conducted in a tertiary care hospital. Patients diagnosed and treated for LABC (>18 years) between January 2011 and June 2019 were selected and those who received NAC and failed to achieve pCR were identified. Patients with incomplete axillary response according to tumor features were also evaluated.

LABC was defined as stage III and stage IIB (T3N0) disease^8^.

Notable exclusions were male patients, patients with metastatic disease at presentation, those who received only neoadjuvant hormonotherapy, and patients with pCR.

Demographic, clinicopathological, and treatment characteristics were recorded. The source of medical records was our hospital's electronic database.

### Pathology

Diagnosis of invasive breast cancer in the breast and axilla was histopathologically confirmed and defined according to WHO classification^9^. ER, PR, and HER2 statuses were evaluated by immunohistochemistry or fluorescence in situ hybridization before NAC administration. Tumor subtypes were defined as follows [12]: luminal A; ER(+) or PR(+), HER2-neu (–), Ki67 <20%; luminal B, ER(+) or PR(+), and/or HER2-neu (+), Ki67 ≥20%; non-luminal HER2-neu(+), ER(–) PR(–) HER2-neu (+); and triple-negative, ER(–) PR (–) HER2-neu (–).

### Treatment

The multidisciplinary team decided on NAC and the type of surgery post-NAC. Most patients received anthracycline-based regimens, followed by a taxane (four cycles of doxorubicin and cyclophosphamide every 3 weeks and 12 cycles of paclitaxel or docetaxel weekly). Patients with HER2-positive tumors received trastuzumab simultaneously with NAC for 1 year^5,10^. No patients received adjuvant chemotherapy. After NAC, patients underwent either BCS or mastectomy. Intraoperative pathological examination was performed to analyze SLNB and surgical margins^11^. Patients with clinically, radiologically, and pathologically positive lymph nodes underwent ALND.

All patients underwent adjuvant radiotherapy. Radiotherapy was applied at 50 Gy in 25 fractions over 4 weeks and 10 Gy boost to the tumor bed for patients who underwent BCS, and peripheral lymph nodes (axillary, supra, and supra infra-clavicular and internal mammary lymph nodes). Patients with ER-positive tumors received adjuvant endocrine therapy with tamoxifen or aromatase inhibitors for at least 5 years^10^.

### Assessment of response

Pathological response after NAC was assessed using the Miller-Payne classification. pCR (ypT0 and ypN0) was defined as no residual invasive disease in the breast or axilla. Residual ductal carcinoma in situ was included in the pCR category. Partial response was defined as any response besides pCR^12^.

### Statistical analysis

The endpoint analyses were LRR, distant metastases, and disease-specific survival (DSS). LRR was defined as a recurrent disease in the ipsilateral breast or peripheral lymph nodes. Distant metastasis was considered any recurrence in distant organs or other lymph nodes.

SPSS Version 22.0 (SPSS Inc., Chicago, IL, USA) was used for statistical analysis. The study data were evaluated using descriptive methods (number, percent, and median). Survival calculations were made by the Kaplan-Meier analysis. With the log-rank univariate analysis test, the effects of prognostic factors related to tumor and patient characteristics on disease-free survival (DFS) and DSS were investigated. The effects of prognostic factors on DFS and DSS were investigated using the Cox regression test in multivariate analysis. Proportional differences between the groups were calculated with the chi-square test. Results were evaluated within a 95% confidence interval and a significance level of p<0.05.

### Ethics

The study protocol was approved by the Istanbul Education and Training Hospital (approval no. 2020-2675). The study was conducted under the 1975 Declaration of Helsinki, as revised in 2013.

## RESULTS

A total of 1,247 patients with breast cancer were diagnosed and treated between January 2011 and June 2019. We identified 205 patients with LABC who received NAC. Among them, 54 (26.3%) achieved pCR in the breast, 94 (45.9%) in the axilla, and 43 (21%) in both breast and axilla. Notably, 151 patients with incomplete response in the breast and 111 with incomplete response in the axilla were included in this study.

Demographic and clinicopathological features of patients with partial response are shown in Table 1. The median age at diagnosis was 52 years (range 25–77 years), and 44% (n=67) were younger than 50 years. Notably, 61 (40%) patients underwent BCS and 90 (60%) underwent mastectomy. As for the axillary approach, 92 (61%) patients underwent ALND and 59 (39%) underwent SLNB.

**Table 1:** Demographic and clinicopathological features of patients who achieved partial response in the breast.

Features	Category	Patients n=151 (%)
Age (years)	<50	67(44.4)
	≥50	84(55.6)
cT stage	I–II	111(73.5)
	III–IV	40(26.5)
cN stage	0–I	130(86.1)
	II–III	21(13.9)
Histological type	Invasive ductal carcinoma	126(83.4)
	Others	25(16.6)
Surgical type	Breast-conserving surgery	61(40.4)
	Mastectomy	90(59.6)
Tumor grade	I–II	106(70.2)
	III	45(29.8)
Lymphovascular invasion	Positive	65(43.0)
In situ carcinoma	Yes	92(60.9)
ypT stage	I	101(66.9)
	II	37(24.5)
	III	13(8.6)
Lymph node invasion	Positive	100(66.2)
Estrogen receptor	Positive	115(76.2)
Progesterone receptor	Positive	96(63.6)
HER2	Positive	33(21.9)
Ki-67	<20%	26(17.2)
	≥20%	125(82.8)
Molecular subtype	Luminal-A	15(9.9)
	Luminal B/HER2(–)	85(56.3)
	Luminal B/HER2(+)	17(11.3)
	Non-luminal B/HER2(+)	16(10.6)
	Triple-negative	18(11.9)
Molecular subtype	Luminal	117(77.5)
	Non-luminal	34(22.5)
Loco-regional recurrence	Breast	0(0.0)
	Thorax wall	6(4.0)
	Axilla	1(0.7)
Systemic recurrence	Yes	20(13.2)

LRFS: Local Recurrence Free Survival, DFS: disease-free survival, DSS: disease-specific survival.

The rate of partial response in the breast was 74%. Evaluation of residual tumors in the breast revealed that 45 (30%) patients had high-grade tumors, 65 (43%) had lymphovascular invasion (LVI), 92 (61%) had coexisting in situ carcinoma, and 100 (66%) had residual tumor in lymph nodes (Table 1).

**Figura 1:**
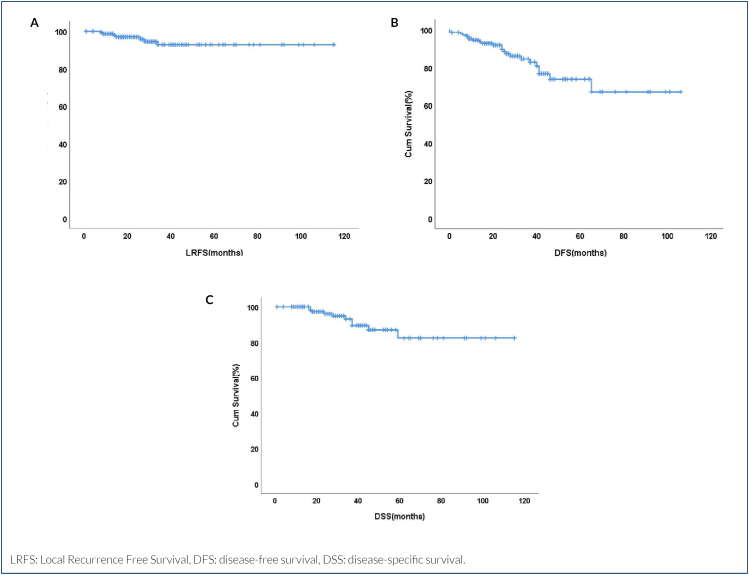
The 5-year locoregional free survival. (A) the 5-year disease-free survival, (B) and the 5-year disease-specific survival and (C) of the patients who failed to achieve pathologic complete response.

After a median of 31 months of follow-up (range 12–115 months), 10 patients (6.6%) died and 20 (13.2%) developed distant metastasis. The 5-year DFS and DSS were 73.8 and 83.1%, respectively (Figures 1B and 1C). Seven patients (4.6%) developed LRR, including six chest wall recurrence and one axillary nodal recurrence. The last LRR occurred in the 34th month. The 5-year locoregional free survival was 92.8%. The nodal failure occurred in a patient who underwent ALND. All chest wall recurrences occurred in patients who underwent mastectomy. LRR rate was significantly higher in patients who underwent mastectomy than patients who underwent BCS (8% vs. 0%; p=0.042, respectively).

On univariable analysis, mastectomy, high-grade tumors, LVI, non-luminal tumors, and HER2 overexpression were associated with LRR. On multivariate analysis, independent factors associated with LRR were LVI [HR: 22.35 (1.42–352.62); p=0.027] and non-luminal molecular subtype [HR: 27.34 (2.99–249.14); p=0.003] (Tables 2 and 3).

**Table 2:** Risk factors associated with 5-year locoregional recurrence-free survival, disease-free survival, and disease-specific survival of patients who achieved partial response in breast (univariate analysis).

Factors	Category	LRRFS (92.8%)	p	DFS (73.8%)	p	DSS (83.1%)	p
Age (years)	<50	91.3	0.485	78.3	0.449	91.2	0.290
	≥50	94.0		70.2		84.6	
cT stage	I–II	93.4	0.932	70.7	0.509	83.7	0.321
	III–IV	90.5		82.0		85.9	
cN stage	0–I	94.3	0.326	75.6	0.421	88.9	0.005*
	II–III	85.5		65.0		69.3	
Histological type	Invasive ductal carcinoma	93.0	0.978	71.8	0.447	81.3	0.219
	Others	91.7		88.0		100.0	
Surgical type	Breast-conserving surgery	100.0	0.040*	86.8	0.044*	96.9	0.108
	Mastectomy	88.9		66.8		77.7	
Tumor grade	I–II	100.0	<0.001*	75.6	0.033*	83.9	0.351
	III	78.4		55.2		79.8	
Lymphovascular invasion	Positive	85.9	0.018*	53.9	<0.001*	62.6	<0.001*
	Negative	97.9		89.1		100.0	
In situ carcinoma	Yes	91.4	0.546	61.5	0.005*	74.3	0.012*
	No	94.9		94.9		100.0	
pT stage	I–II	92.8	0.754	77.0	0.007*	86.8	0.034*
	III	91.7		53.3		77.1	
Lymph node invasion	Yes	89.4	0.058	68.5	0.021*	75.1	0.024*
	No	100.0		82.6		100.0	
Estrogen receptor	Positive	97.3	0.002*	73.5	0.235	83.1	0.325
	Negative	78.8		70.1		81.4	
Progesterone receptor	Positive	98.7	0.005*	74.5	0.169	82.0	0.444
	Negative	82.4		71.1		83.0	
HER2	Positive	81.7	0.003*	77.7	0.590	93.0	0.567
	Negative	96.5		71.7		78.8	
Ki-67	<20%	100.0	0.239	87.5	0.110	100.0	0.659
	≥20%	91.5		71.4		88.2	
Molecular subtype	Luminal-A	100.0	0.002*	83.3	0.507	100.0	0.637
	Luminal B/HER2(–)	98.5		75.2		81.0	
	Luminal B/HER2(+)	91.7		91.7		100.0	
	Non-luminal B/HER2(+)	71.3		63.2		85.7	
	Triple-negative	80.0		72.9		68.6	
Molecular subtype	Luminal	97.3	0.001*	74.3	0.153	83.4	0.259
	Non-luminal	77.4		67.8		80.1	

* p<0.05, p-value given refer to log-rank test. DFS: disease-free survival; DSS: disease specific survival; LRRFS: locoregional recurrence free survival.

**Table 3:** Risk factors associated with 5-year locoregional recurrence free survival, disease free survival and disease specific survival of patients who achieved partial response in breast (multivariate Cox regression analysis).

Factors	Category	Loco-regional recurrence-free survival HR (95%CI)	p-value	Disease-free survival HR (95%CI)	p-value	Disease-specific survival HR (95%CI)	p-value
Age (years)	<50	3.55(0.44–28.94)	0.237	Reference (1)	0.617	Reference (1)	0.125
	≥50	Reference (1)		1.30(0.46–3.65)		3.41(0.71–16.34)	
cT stage	I–II	Reference (1)	0.135	Reference (1)	0.003*	Reference (1)	0.358
	III–IV	5.65(0.58–54.88)		5.66(1.77–18.09)		2.06(0.44–9.62)	
cN stage	0–I	Reference (1)	0.791	Reference (1)	0.803	Reference (1)	0.025*
	II–III	1.34(0.15–11.87)		1.14(0.40–3.27)		5.14(1.22–21.59)	
Histological type	Invasive ductal carcinoma	Reference (1)	0.237	Reference (1)	0.607	Reference (1)	
	Others	5.66(0.39–82.06)		1.49(0.32–6.88)		**	
Surgery	Breast-conserving surgery	Reference (1)		Reference (1)	0.444	Reference (1)	0.200
	Mastectomy	**		1.67(0.45–6.17)		4.23(0.47–38.44)	
Tumor grade	I–II	Reference (1)		Reference (1)	0.180	Reference (1)	0.941
	III	**		2.02(0.72–5.64)		1.06(0.24–4.68)	
Lymphovascular invasion	Positive	22.35(1.42–352.62)	0.027*	4.35(1.18–15.94)	0.027*	**	
	Negative	Reference (1)		Reference (1)		Reference (1)	
In situ carcinoma	Yes	1.04(0.15–6.98)	0.972	7.37(1.52–35.71)	0.013*	**	
	No	Reference (1)		Reference (1)		Reference (1)	
pT stage	I–II	Reference (1)	0.499	Reference (1)	0.004*	Reference (1)	0.089
	III	2.58(0.17–40.34)		5.42(1.69–17.35)		3.87(0.81–18.45)	
Lymph node invasion	Yes	**		3.70(0.77–17.87)	0.103	**	
	No	Reference (1)		Reference (1)		Reference (1)	
Molecular subtype	Luminal	Reference (1)	0.003*	Reference (1)	0.015*	Reference (1)	0.233
	Non-luminal	27.34(2.99–249.14)		4.41(1.33–14.58)		2.75(0.52–14.55)	

* p<0.05; hazard ratio (HR) is presented with their 95% confidence interval (CI) and p-value.

** As there is no event, it is not included in Cox regression modeling.

As for 5-year DFS rates, patients who underwent mastectomy with high-grade tumors, LVI, residual yp TIII, residual in situ carcinoma, and residual tumor in lymph nodes had lower DFS rates than those without these features. Independent factors affecting DFS were LVI [HR: 4.35 (1.18–15.94); p=0.027], residual in situ carcinoma [HR: 7.37 (1.52–35.71); p=0.013], yp TIII stage [HR: 5.42 (1.69–17.35); p=0.004], and non-luminal molecular subtype [HR: 4.41 (1.33–14.58); p=0.015]. We observed that in cases of partial axillary response, higher numbers of metastatic lymph nodes were associated with lower 5-year DFS [(1–3 involved lymph nodes: 81%, 4–9: 70.4% and ≥10: 46.7%) (p=0.025)] (Tables 2–4). However, metastatic tumor size and axillary extranodal extension (ENE) were not associated with survival.

**Table 4:** Results of 5-year disease-free survival and overall survival related to axillary lymph node status (number of positive lymph nodes, size of the metastasis, and extranodal extension) of patients with axillary partial response.

Factors	Category	DFS (67.8%)	p	DSS (75.8%)	p
Number of metastatic lymph nodes	1–3	81.0	0.025*	92.1	
	4–9	70.4		81.8	
	≥10	46.7		62.5	0.453
Size of metastasis	ITC and micrometastasis	79.1	0.421	90.0	
	Macrometastasis	65.3		73.4	0.603
Extra nodal extension	Yes	62.5	0.385	68.0	
	No	76.5		93.8	0.148

* p<0.05, p-values given refer to log-rank test. DFS: disease-free survival; DSS: disease-specific survival; ITC: isolated tumor cells.

DSS rate of patients with cNII-III stages, LVI, coexisting in situ carcinoma, ypT III stage, and positive lymph nodes were significantly lower than those without these features. We determined that the only independent factor affecting 5-year DSS was cN II–III stage [HR: 5.14 (1.22–21.59; p=0.025] (Tables 2 and 3).

## DISCUSSION

This retrospective study evaluates predictive factors for recurrence in LABC patients without pCR after NAC. We found that LVI and non-luminal subtypes were independent predictors of LRR. LVI, in situ carcinoma, yp TIII stages, and non-luminal molecular subtypes were independent predictors affecting DFS. The only independent factor affecting DSS was cN II–III. Our results demonstrate that the number of involved axillary lymph nodes were independent predictors of LRR and DSS for patients without axillary pCR.

Patients with residual disease post-NAC have a higher risk of developing LRR than patients with pCR^3,12,13^.

In many studies, the presence of LVI increases recurrence risk in patients without pCR^13,14^. Our results support this by showing that LVI was a predictor of LRR for patients without axillary pCR.

ENE, which is defined as an extension of neoplastic cells through the nodal capsule into perinodal tissue, is associated with recurrence and mortality. Tumors with ENE persisting after NAC may be more likely to recur^15^. Unlike the reported literature, we did not observe any relationship between ENE and survival in patients without axillary pCR.

Although patients with luminal-type, especially hormone-positive, or non-HER2-like tumors are less likely to achieve pCR than patients with non-luminal and HER2-like tumors, they are not necessarily more likely to develop LRR^3^. In fact, studies show that patients with TNBC with residual disease after NAC have a higher recurrence risk^4,13^. Supporting literature, this study shows that hormone-positive tumors, especially luminal B-type tumors, were less likely to achieve pCR than non-luminal tumors. However, the non-luminal subtype was an independent predictor of local or systemic recurrences.

In patients with residual disease post-NAC, lymph node positivity and LVI are predictors of distant metastasis^13,14^. The only nodal failure in this study occurred in the axilla of a patient who underwent ALND. Similarly, all chest wall recurrences occurred among patients who underwent mastectomy, and no in-breast failure occurred among patients who underwent BCS. Based on this, we concluded that more extensive surgeries result in more aggressive tumors. This study also showed that high numbers of involved lymph nodes are predictors of axillary recurrence. Extensive axillary dissections showed no additional benefit in avoiding LRR. As no relapses occurred after SLNB, ALND may be less considered after NAC and SLNB may be preferred even for patients with a partial response.

Our study has several limitations. First, data were collected retrospectively. Second, no formal power analysis was performed to determine the sample size. However, all eligible patients with LABC in our hospital database were recruited. Third, this is a single-center study and does not contain different therapeutic approaches. Therefore, the generalizability of our results may be limited due to varying treatment guidelines according to local regulations and resource availability.

In conclusion, our study demonstrated that LVI and non-luminal subtypes are independent predictors of LRR. LVI, in situ carcinoma, ypTIII stage, and non-luminal molecular subtypes are independent predictors of DFS. The only independent factor affecting DSS was cN II-III. Additionally, the number of involved axillary lymph nodes was an independent predictor of LRR and DSS. Residual tumor size and ENE did not demonstrate a significant risk for recurrence in patients without axillary pCR. As patients with residual disease are prone to recurrence, identifying these patients is essential for monitoring and early intervention. Our study also showed that BCS and SLNB are safe after NAC, as no recurrence occurred in patients who underwent these procedures. Patients with non-luminal large residual tumors in the breast and axilla, with in situ carcinoma or LVI, may benefit from adjuvant chemotherapy with agents that sensitize tumors to chemotherapy and radiotherapy to lower recurrence risk. Future prospective, powerful, and genetic-based studies are warranted.
